# Mothers' knowledge, practice, and attitudes toward vitamin D deficiency among children in the Qassim region, Kingdom of Saudi Arabia

**DOI:** 10.25122/jml-2021-0384

**Published:** 2022-09

**Authors:** Samah El Awady Bassam, Fawzia Nabeel Mohammad Abd-Elmageed

**Affiliations:** 1 Department of Maternal and Child Health Nursing, College of Nursing, Qassim University, Qassim, Kingdom of Saudi Arabia; 2 Department of Pediatric Nursing, Faculty of Nursing, Zagazig University, Zagazig, Egypt

**Keywords:** attitude, knowledge practice, vitamin D deficiency

## Abstract

Vitamin D comes in two forms: ergocalciferol (D2) and cholecalciferol (D3). This study aimed to assess mothers' knowledge, attitudes, and practices toward vitamin D deficiency among children. We collected data using a self-administered online questionnaire to gather information about the characteristics, knowledge, attitudes, and reported practices of 800 Saudi Arabian mothers regarding vitamin D deficiency. The mean age of participants was 31.2±4.76, and 8% had a university education. When it came to household income, the majority (90.1%) reported that they had enough money. Participants who attended training courses, employed mothers, total practice, and total attitude had a significant favorable effect on knowledge, with a p-value of less than 0.01. Employed mothers, training course attendees, total practice, and overall attitude had a significant favorable effect on mothers' practice, with a p-value of less than 0.01**. More than half of the mothers who participated in the study had an inadequate level of understanding. Less than two-thirds of those surveyed noted the deficient practice. Two-thirds of the moms who participated in the study had a negative attitude toward vitamin D deficiency. There was a strong positive correlation between total knowledge, total attitude, and total practice-related vitamin D deficiency.

## INTRODUCTION

Calcium and phosphorus metabolism is regulated by vitamin D, a fat-soluble vitamin. Vitamin D2 and D3 are the two forms of vitamin D. Two micronutrients essential for bone health, muscle contraction, neuron excitability, and blood coagulation are calcium and vitamin D. Vitamin D modulates bone and intestine absorption to regulate calcium plasmatic concentration [1–3].

Sunlight is the principal source of vitamin D. Oily fish like sardines, tuna, salmon, mackerel, egg yolks, cod liver oil, and mushrooms are all excellent sources of vitamin D [4]. Compared to animal milk, human milk is deficient in vitamin D. Hypocalcaemia seizures or convulsions may be caused by vitamin D insufficiency, especially in newborns and adolescents growing rapidly. Bone deformities can be seen in infants as young as six months old if vitamin D insufficiency is present (rickets).

In addition to irritability and weight-bearing issues, children with vitamin D deficiency may also show stunted growth, be more susceptible to infections, have less lung expansion, and have weaker muscles. Too little vitamin D can cause cardiomyopathy, leading to heart failure and even death [5, 6].

As the incidence of vitamin D deficiency in children rises, so does the need for mothers to receive additional training. An infant's health is closely linked to the mother's education, as she is the primary caregiver for her child. Therefore, it is important to help mothers understand the possible causes of the decline in the levels of these important substances. Despite one's daily dietary patterns, how much UVB exposure and supplementation may be needed [7] is critical to understanding vitamin D levels. Pediatric nurses play a critical role in educating children about the dangers of vitamin D deficiency. Educating themselves and others about vitamin D deficiency (VDD) is a critical first step for nurses to increase knowledge and prevention. Educating women on the need for sun exposure and the sources of vitamin D could be done by nurses. Vitamin D deficiency can be prevented by exposing children to sunlight and providing adequate vitamin D supplements. Mothers are responsible for the majority of their children's nutritional needs. The nutritional content of children's diets is directly influenced by their mothers' awareness of healthy eating habits. Our goal was to develop and implement an educational program to close the gap in maternal knowledge, practice, and attitude regarding vitamin D and raise awareness about the critical role that vitamin D plays in their children's health. It is necessary to evaluate maternal knowledge, practice, and attitude regarding vitamin D and identify gaps in their learning, training, and attitude. To meet the purpose of the study, we aimed to assess the level of knowledge, practice and attitudes of mothers toward vitamin D insufficiency among their children [8–9].

## MATERIAL AND METHODS

This descriptive cross-sectional study was performed in different cities in the Al-Qassim region, including Buraydah, Al-Rass, Unaizah, Al-Bada'a, Almuthnab, and other areas.

### Participants

Eighty hundred women participated (n=800), with a 5% α error with 95% significance and a 20% β error with 80% significance. The sample size was estimated using the MedCalc statistical software tool (http://www.medcalc.org/index.php). The research survey was conducted in the Saudi Arabia Kingdom, in Qassim City, from 10^th^ June 2021 to 20^th^ October 2021.

### Data collection and pilot study

The data collection tool for this cross-sectional study was a self-administered online survey used to verify compliance with current public health standards, such as social distance and limiting one-on-one encounters. Mothers of children under five were invited to participate in the survey by completing an electronic questionnaire. The online survey was administered through Google Forms and sent to the mothers through social media platforms such as Facebook, WhatsApp, and mailing lists. The validated questionnaire was pre-tested on 80 moms (10% of the total) who were later enrolled in the main research. The following three sections of a validated Arabic-language questionnaire were utilized for data collection.

Part I: Sociodemographic characteristics (age, educational level, occupation, number of children, children under five years, training courses etc).

Part II: Women's knowledge regarding vitamin D insufficiency.

This tool was adapted from Zadka et al. [10] and translated into Arabic to measure women's knowledge regarding vitamin D deficiency (including 12 multiple questions such as sources of vitamin D, causes of vitamin D deficiency, signs and symptoms, complications of vitamin D deficiency etc). Responses were scored as 1 for correct answers and zero for incorrect. A total score of ≥70% was regarded as sufficient knowledge and <70% as insufficient.

Part III: Women's reported practice related to children's vitamin D insufficiency.

This tool was adapted from Selim et al. [11] and translated into Arabic to measure women's practice regarding vitamin D deficiency (including 12 multiple questions such as feeding, vitamin D supplements, sun exposure etc). Responses were scored as 1 for correctly done and zero for not doing. A total score of ≥70% was regarded as satisfactory practice and <70% as unsatisfactory.

Part IV: Women's attitudes towards vitamin D deficiency in children.

This tool was adapted from Zadka et al., 2018 [12] and translated into Arabic to measure mothers' attitudes toward vitamin D deficiency. It consisted of eight items. Each item was rated on a three-point Likert scale ranging from 1 (agree), 2 (neutral), and 3 (disagree). To sum up, higher scores indicated a higher altitude. As a result, the overall attitude score was divided into the following two categories: a positive attitude (≥70%) and a negative attitude (<70.0%).

### Reliability

Cronbach's alpha coefficient test in SPSS version 24 was used to determine the reliability of the customized questionnaire. The online questionnaire's internal consistency reliability (Cronbach's) was good at 0.855.

### Statistical analysis

Data were sorted and categorized, and the findings were shown in table format. Data were analyzed using the Statistical Package for the Social Sciences (SPSS) software (SPSS Inc; version 21; IBM Corp., Armonk, NY, USA). The Kolmogorov-Smirnov test was used to evaluate the normality of the data in a single sample. When describing qualitative data, numbers and percentages were employed. A scalar response and one or more explanatory factors were modeled using linear regression, provided as means plus standard deviation and n. Scalar responses were modeled utilizing a scalar response and one or more explanatory variables. A p-value of 0.05 was regarded as statistically significant.

## RESULTS

Table 1 presents the mothers' sociodemographic characteristics. The mean age was 31.2±4.76, and more than half (53.8%) had a university education. Regarding family income, the majority (90.1%) had sufficient income. More than half (62.4%) of mothers were housewives, and nearly two-thirds (64%) had one child under five.

**Table 1:** Participants characteristics (n=800).

Items	N	%
Age		
20–25	150	18.8
25–30	177	22.1
30–35	240	30
35 or more	233	29.1
Mean SD 31.2±4.76		
Education level		
Preparatory	123	15.4
Secondary	246	30.8
University	431	53.8
Family income		
Insufficient	79	9.9
Sufficient	721	90.1
Occupational status		
Employed	301	37.6
Housewife	499	62.4
Number of children		
1	74	9.2
2	126	15.7
3	320	40.1
>3	280	35
Number of children less than five years		
1	512	64
2	199	24.9
3	76	9.5
>3	13	1.6
Training courses about Vit D deficiency		
Yes	82	10.3
No	718	89.7

**Figure 1:**
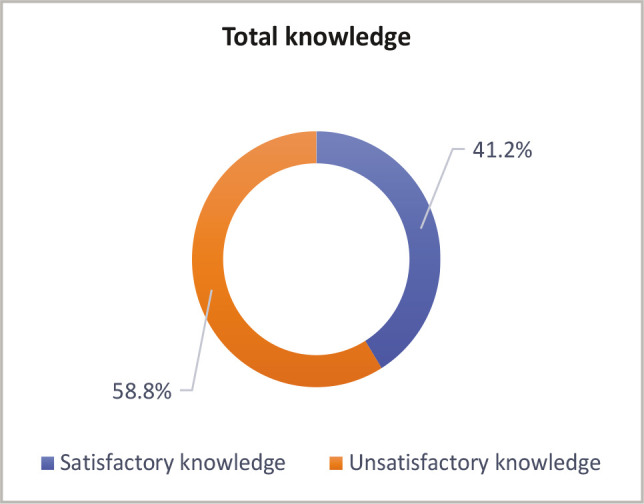
Distribution of mothers according to their total knowledge about vitamin D deficiency (n=800).

Around two-fifths (41.20%) of the mothers surveyed were aware of the dangers of vitamin D insufficiency. However, more than half (58.8%) had insufficient knowledge (Figure 1).

**Figure 2:**
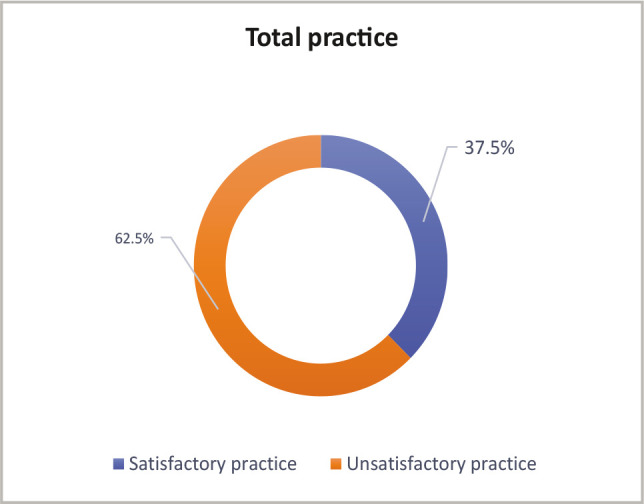
Distribution of mothers according to their total practice regarding vitamin D (n=800).

More than one-third (37.5%) of mothers had good practices related to vitamin D, however, less than two-thirds (62.5%) reported an unsatisfactory approach (Figure 2).

**Figure 3:**
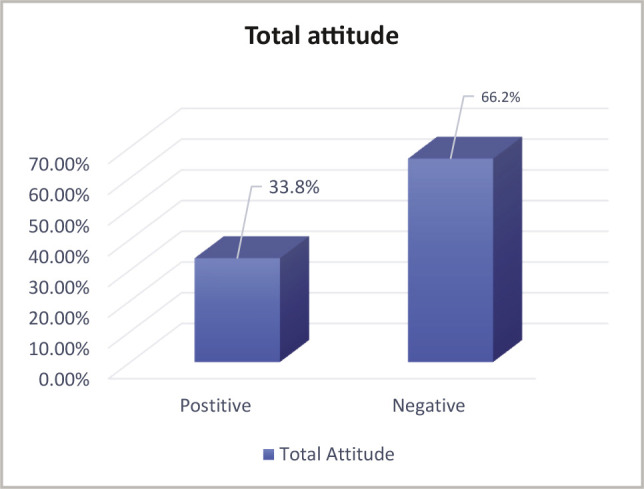
Distribution of mothers according to their total attitude towards vitamin D deficiency (n=800).

Two-thirds (66.2%) of mothers had a negative attitude towards vitamin D deficiency, however, one-third (33.8%) had a positive attitude (Figure 3).

Table 2 revealed that the model is highly significant (F=9.023, P-value=0.000**). This model explains 56% of the variation in the total knowledge of mothers detected through an R^2^ value of 0.56. Attending training courses, being employed, total practice and total attitude had a high positive effect on mothers' knowledge (p-value<0.01), while university education and sufficient family income had a slightly positive impact on mothers' knowledge (p-value<0.05).

**Table 2:** Linear regression model for the total knowledge level.

	Unstandardized Coefficients B	Standardized Coefficients β	T-test	P-value
**Education level (University)**	.146	.265	2.771	.041*
**Family income (Sufficient)**	.197	.301	4.976	.016*
**Training courses (Yes)**	.345	.487	7.080	.009**
**Occupation status (Employee)**	.364	.499	8.665	.006**
**Total attitude**	.278	.390	6.967	.009**
**Total practice**	.301	.400	7.560	.007**
**ANOVA**				
Model	R^2^	F	P-value	
Regression	0.56	9.023	.000**	

a. Dependent Variable: Total knowledge; b. Predictors: (constant): education level (university), family income (sufficient), training courses (yes), occupation status (employee), total attitude, and total practice.

Table 3 revealed that the model is highly significant (F=10.765, P-value=0.001**), which explains 61% of the variation in the total practice of mothers detected through R^2^ value 0.61. Also, attending training courses, being employed and total attitude had a high positive effect on mothers' practice (p-value<0.01)

**Table 3:** Linear regression model for total practice level.

	Unstandardized Coefficients B	Standardized Coefficients β	T-test	P-value
**Age**	.218	.416	3.164	.019*
**Education level (University)**	.301	.397	8.011	.005**
**Had children less than five years (>2)**	.182	.297	3.998	.017*
**Training courses (Yes)**	.311	.423	6.723	.008**
**Total attitude**	.267	.325	6.808	.008**
**Total knowledge**	.386	.449	9.012	.003**
**ANOVA**				
Model	R^2^	F	P-value	
Regression	0.61	10.765	.001**	

a. Dependent Variable: Total practice; b. Predictors: (constant): age, education level (university), had children less than five years (>2), training courses (yes), total attitude, and total knowledge.

Table 4 shows a strong positive correlation between total knowledge, attitude, and practice concerning vitamin D deficiency (p=0.01).

**Table 4:** Correlation between studied variables.

	Total practice	Total attitude
**Total knowledge**	R 0.567 P<0.01**	R 0.499 P<0.01**
**Total practice**		R 0.423 P<0.01**

## DISCUSSION

Less than one-third of the mothers' ages ranged between 30 to less than 35 years. More than half had a university education, and most had sufficient income. Less than two-thirds were housewives and had one child less than five years. Additionally, most mothers did not attend training courses about vitamin D, which matched with other studies [13, 14]. On the other hand, this outcome disagrees with another study [15] which showed that almost half of the mothers were employed. The study found that more than half of the studied mothers had insufficient knowledge regarding vitamin D deficiency regarding total knowledge scores. This outcome agrees with Zadka et al., 2018 [16], who revealed that more than half (56.0%) of the mothers who participated in the study were unaware of the dangers of vitamin D insufficiency. According to the researcher, the absence of healthcare services may have contributed to this outcome. Workers' educational responsibilities include educating and informing mothers about the need for diet and vitamins for the appropriate development of their children. Another study in Saudi Arabia by Alwadei et al. [17] found that the highest percentage of females with studies had good knowledge regarding vitamin D deficiency. This study revealed that less than two-thirds of the mothers had unsatisfactory practice for vitamin D deficiency which agrees with Bezabih et al. [18]. This result might be due to a lack of knowledge and awareness of the mothers regarding the importance of vitamin D.

Our study indicated that less than two-thirds of the mothers had a negative attitude. This outcome agrees with Ciçek et al. [19], who conducted a study on the mother's level of knowledge and attitudes regarding vitamin D use in Konya, where the majority had a negative attitude about vitamin D deficiency. Moreover, this result is supported by another study [20], which proved that most mothers had a negative attitude regarding vitamin D. The current result revealed that complete knowledge of mothers affected by attending training courses, employment, total practice, total attitude had a high positive effect on mothers' knowledge at p-value<0.01**, while university education and sufficient family income had a slight positive effect on mothers' knowledge with p-value<0.05*. This outcome is consistent with another study [21], which showed that age and education had a slight positive effect on mothers' knowledge. Moreover, this result agrees with another study [9] which found a strong association between total knowledge score and age, level of education, and socioeconomic status. The current study revealed that attended training courses, employee mothers, total practice, and total attitude positively affected mothers' knowledge at p-values<0.01**. In contrast, university education and sufficient family income slightly affected mothers' practice (p-value<0.05*).

As a result, this finding conflicts with another study [22] that discovered no significant statistical relationship between mothers' practice regarding vitamin D deficiency and their Sociodemographic characteristics (mothers' age, residence, type of family, number of children, occupation, level of education, and monthly income). Total knowledge, attitude, and total vitamin D insufficiency in practice were all positively correlated, with a p-value of 0.01**. This result disagrees with Abdel Nabi et al. [23], who stated a positive but not statistically significant correlation between total knowledge & total practice of females regarding vitamin D deficiency. According to the study, this outcome might be attributed to a rise in information, which leads to an increase in attitudes and behaviors towards vitamin D shortage, which directly leads to good practices in dealing with the health problem linked with vitamin D deficiency.

### Recommendations

Based on the results of the current study, the following recommendations are suggested: Nurses at family centers must take on additional responsibility for maintaining and spreading education and training on the significance of vitamin D among women who visit them during pregnancy and after childbirth. Mothers should introduce complementary foods to their children's diets at 6 to 12 months, according to Polish infant nutrition guidelines. To enhance the possibility of optimal vitamin D levels in their kids, women should be supplied with healthy food and lifestyle throughout the early stages of pregnancy.

Vitamin D insufficiency can be minimized by providing mothers with enough assistance and advice, such as giving supplementation and education on the necessity of adequate vitamin D consumption. To avoid vitamin D insufficiency, healthcare practitioners must engage in educational programs on the proper beginning and maintenance of vitamin D doses. Programs on vitamin D health education should be offered regularly at appropriate phases throughout pregnancy and after birth. Healthcare practitioners must be educated on the necessity of vitamin D supplementation and the signs and symptoms that indicate it should be discontinued.

## CONCLUSION

More than half of the mothers included in the study had insufficient knowledge, less than two-thirds reported deficient practice, and two-thirds held a negative attitude towards vitamin D insufficiency. There was also a significant positive relationship between total knowledge, attitude, and practice-related vitamin D deficits.

## Personal thanks

We would like to express our gratitude to all mothers who participated in this investigation.
